# Tracking genome-editing and associated molecular perturbations by SWATH mass spectrometry

**DOI:** 10.1038/s41598-019-51612-z

**Published:** 2019-10-23

**Authors:** Qifeng Lin, Larry W. L. Low, Adam Lau, Esther W. L. Chua, Yuji Matsuoka, Yilong Lian, Antónia Monteiro, Stephen Tate, Jayantha Gunaratne, Tom J. Carney

**Affiliations:** 1 Institute of Molecular and Cell Biology, Agency for Science, Technology and Research, Singapore, 138673 Singapore; 2 SCIEX, Concord, Ontario L4K 4V8 Canada; 3 Department of Biological Sciences, National University of Singapore, 117543 Singapore, Singapore; 4 Yale-NUS College, 138609 Singapore, Singapore; 5 Yong Loo Lin School of Medicine, National University of Singapore, Singapore, 117594 Singapore; 6 Lee Kong Chian School of Medicine, Nanyang Technological University, Singapore, 636921 Singapore

**Keywords:** Mass spectrometry, Gene targeting

## Abstract

Advances in gene editing now allow reverse genetics to be applied to a broad range of biological systems. Ultimately, any modification to coding sequences requires confirmation at the protein level, although immunoblotting is often hampered by antibody quality or availability especially in non-model species. Sequential Window Acquisition of All Theoretical Spectra (SWATH), a mass spectrometry (MS) technology with exceptional quantitative reproducibility and accuracy, offers an ideal alternative for protein-based confirmation. Here, using genome edits in mouse, zebrafish and *Bicyclus anynana* butterflies produced using either homologous recombination or targeted nucleases, we demonstrate absence of the targeted proteins using SWATH, thus confirming successful editing. We show that SWATH is a robust antibody-independent alternative for monitoring gene editing at the protein level and broadly applicable across diverse organisms and targeted genome manipulation techniques. Moreover, SWATH concomitantly defines the global proteome response in the edited organism, which may provide pertinent biological insights.

## Introduction

The ability to introduce targeted changes to the genome has facilitated investigation of gene function and disease modelling in increasingly diverse systems. Strategies for genome manipulation include homologous recombination and targeted nucleases such as zinc finger nuclease (ZFNs) and Clustered, Regularly Interspaced, Short Palindromic Repeat (CRISPR) technologies^1^. As the genetic lesion induced by nuclease-mediated editing is mostly repaired by non-homologous end joining, the size of the indel generated is unpredictable. Thus, it is essential to characterise the induced genetic lesion through genomic DNA sequencing to predict the subsequent coding region frameshift. However, recent observations in CRISPR-knockout experiments have shown that unanticipated outcomes such as alternate translation start sites or exon skipping may result in expression of unexpected protein products which retain function and obviate indel frame-shifts^2^. This phenomenon has been observed in cultured mammalian cells^3,4^, zebrafish^5^, and butterflies^6^.

Whilst RT-PCR experiments or RNAseq might identify transcript variants avoiding genetic lesions, a more direct validation of mutagenesis is to analyse the targeted protein using antibodies, especially in cases when there is no obvious phenotype. However, antibodies are not available for all proteins, especially for non-model organisms with limited or non-existent antibody resources. Indeed, CRISPR technology has arguably the greatest impact on non-model organisms by offering simple and cheap reverse genetics. Using studies in mouse mammary gland epithelial cells, zebrafish embryos and adult fins, and butterfly pupal wings, we show that SWATH-MS^7^, a data-independent acquisition (DIA) technique with superior data reproducibility and accuracy, is a straightforward, rapid and robust method for verifying genome editing at the protein level. Moreover, SWATH simultaneously provides an overview of the associated proteome perturbations, enables *post hoc* data re-interrogation, as well as library-independent validation of protein targeting in non-model organisms.

## Results and Discussion

**Figure 1:**
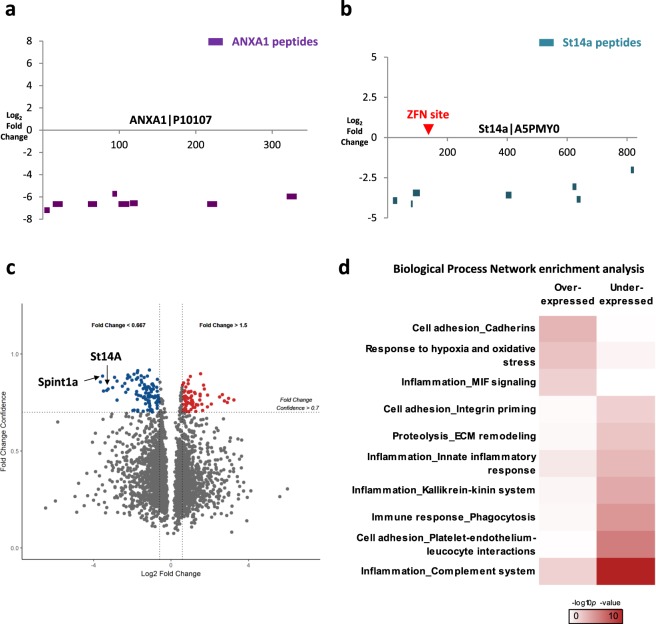
SWATH detects protein silencing and accompanying proteome response. (**a**) ANXA1 peptides (indicated by purple horizontal bars) from SWATH comparison of ANXA1^*−/−*^ vs. ANXA1^*+/−*^ samples presented with their quantified fold changes (y-axis) and respective positions on the ANXA1 protein sequence (x-axis, numbers represent amino acid residue numbers). Expression of all quantified ANXA1 peptides was considerably lower in the ANXA1^*−/−*^ samples, confirming ANXA1 protein knockout. (**b**) A premature termination codon was introduced using zinc finger nuclease (ZFN) in the *st14a* gene (site indicated by red arrow). Similarly, quantified St14a peptides (indicated by turquoise horizontal bars) observed to be in substantially lower levels in the *st14a*^*sq10/sq10*^ mutant adult fin samples, indicating loss of St14a protein. (**c**) Volcano plot visualisation of accompanying proteome changes in response to loss of St14a, showing 73 over-expressed and 98 under-expressed proteins with cut-off criteria of fold change >1.5 and fold change confidence >0.7. (**d**) Top ten biological process networks which show significant enrichment in differentially expressed proteins in the *st14a* mutants compared to siblings.

We assessed a previously characterised primary mammary gland epithelial cell line established from mice, in which exons 3 and 4 of the *Annexin-1* (*Anxa1*) gene were replaced with LacZ and PGKneo by homologous recombination insertion^8,9^. SWATH analysis on *Anxa1*^*+/−*^ and *Anxa1*^*−/−*^ cells showed that levels of all quantified ANXA1 peptides were expectedly lower in *ANXA1*^*−/−*^ cells (Fig. 1a). This result corresponded with antibody-based validation (Supplementary Fig. S1a), illustrating that SWATH can be used to demonstrate successful gene editing. In order to distinguish between proteins which were altered in abundance in response to the ANXA1 silencing from experimental and biological noise, we used several statistical cut-off criteria: requiring at least two unique peptides quantified per protein, fold change >1.5 and fold change confidence >0.7. The fold change confidence is a measure of the confidence that there is a change between the different experimental conditions. This is a combination of the reproducibility and signal quality of the quantified peptides which were used to calculate the fold change as well as the magnitude of the change^10^. Using these criteria, a total of 467 proteins were determined to be differentially expressed. Functional analysis of these differentially expressed proteins indicated enrichment of adhesion and extracellular matrix (ECM) remodelling-related processes (Supplementary Fig. S1b,c), consistent with previous observations^9^ and thus making it highly probable that the changes in the abundances of these proteins are due to the absence of the ANXA1 protein.

Subsequently, we applied this approach to confirm mutagenesis in other models where antibodies were unavailable. We investigated a mutant where a 5 bp insertion was introduced to the 6th exon of the zebrafish *suppressor of tumourigenicity 14a* (*st14a, matriptase1a*) gene using Zinc Fingers (c.465_466insTCACA). This resulted in a predicted frame shift (p.Ala156SerfsTer9), truncating the protein at 163 amino acids from a wild type protein of 834 amino acids. Comparative SWATH analysis of adult fin clips samples showed that all quantified St14a peptides were in considerably lower levels in the *st14a*^*−/−*^ mutant compared to wild type (Fig. 1b). Considering the quantitative reproducibility and accuracy of SWATH which is comparable to selected reaction monitoring^7^, and the consistent under-expression of all St14a peptides, the St14a protein is highly likely to be absent in the mutant. Together with sequencing evidence that confirms successful mutation at the genomic DNA level, our analysis provides proof that the ZFN-induced lesion indeed resulted in loss of St14a protein. We also noticed loss of St14a peptides both upstream and downstream of the ZFN site in the mutant, suggesting the frameshift triggered nonsense-mediated mRNA decay^11^, or the generated protein was unstable or degraded.

St14 (Matriptase1) is a transmembrane serine protease that targets several substrates, including ECM components, other proteases of proteolytic cascades such as uPA, and signalling receptors such as PAR2 and c-Met^12,13^. St14 dysregulation increases cell invasiveness and inflammation^14^, and its activity is restrained through binding a cognate inhibitor, Spint1 (Serine Peptidase Inhibitor Kunitz Type 1)^15^. Spint1, through restricting St14a activity, is essential for maintaining epithelial integrity in zebrafish and mice^14,16^, whilst loss of Matriptase1 in mice results in defective epidermal barrier formation and reduction of Kallikrein-mediated inflammation and corneodesmosome degradation^17,18^. Although overt barrier defects were not apparent in the zebrafish *st14a*^*−/−*^ mutants, we observed relevant functional processes in the 171 differentially expressed proteins (Fig. 1c), including ECM remodelling and Kallikrein-mediated inflammatory responses (Fig. 1d). Interestingly, the complement system, which is downstream of the uPA-plasminogen activation cascade^19^, also appears to be reduced in the absence of St14a. In addition, one of the most under-expressed proteins in the *st14a*^*−/−*^ mutant was the cognate inhibitor, Spint1a itself. Whilst it is well appreciated that St14 and Spint1 form a strong complex within the cell, at the membrane and upon shedding^20–22^, it is surprising that there is a tissue-level reduction of Spint1a protein levels upon loss of St14a protein, and may indicate reciprocal stabilisation of the complex. Similar to the previous ANXA1 model, although changes in levels of other proteins could be due to off-target effects of gene editing, we are unable to distinguish these from differential regulation. However, their association with processes previously related to the function of ST14 strongly suggest that majority of these proteins are changing in response to the absence of the targeted protein. SWATH thus can demonstrate successful nuclease-mediated gene targeting at the protein level and associated global proteome responses in a single experiment.

**Figure 2:**
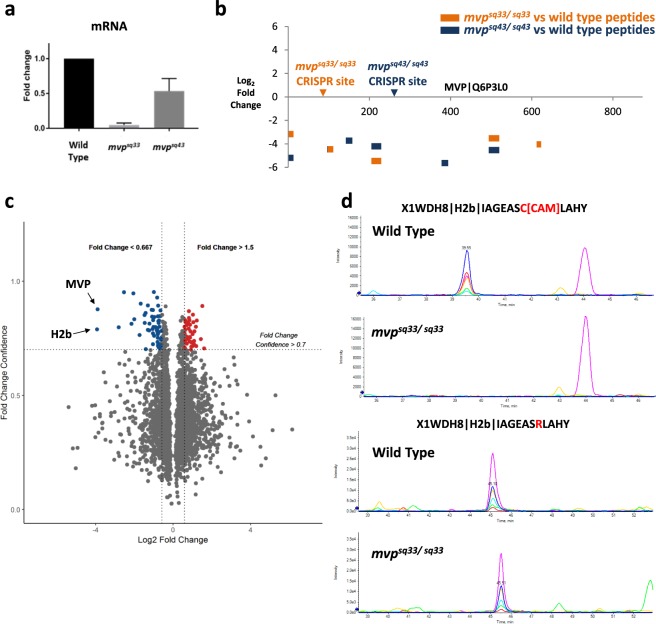
SWATH enables data re-interrogation without need for data re-acquisition. (**a**) Quantitative PCR analysis of *mvp* transcript levels in *mvp*^*sq33/sq33*^ and *mvp*^*sq43/sq43*^ mutants. (**b**) Locations of the predicted termination location of the CRISPR-induced frame shifts in the *mvp*^*sq33/sq33*^ (orange arrow) and *mvp*^*sq43*^ (blue arrow) mutant lines. Resulting MVP peptides (indicated by orange horizontal bars for *mvp*^*sq33/sq33*^ and blue horizontal bars for *mvp*^*sq43/sq43*^) observed to be in substantially lower levels in the mutant samples, indicating loss of MVP protein. (**c**) Volcano plot visualisation of accompanying proteome changes in response to loss of MVP in the *mvp*^*sq33/sq33*^ mutant, showing 42 over-expressed and 59 under-expressed proteins with cut-off criteria of fold change >1.5 and fold change confidence >0.7. The Histone H2b (X1WDH8) protein showed the largest extent of under-expression in the *mvp*^*sq33/sq33*^ mutant. (**d**) Representative extracted ion chromatograms (XICs) of canonical H2b peptide (IAGEASCLAHY) in wild type and *mvp*^*sq33/sq33*^ samples suggested H2b absence in the *mvp*^*sq33/sq33*^ mutant. However, re-extraction of SNP-variant peptide (IAGEASRLAHY) revealed presence of H2b in *mvp*^*sq33/sq33*^ samples.

Next, we assessed if SWATH could similarly detect zebrafish gene mutations introduced by CRISPR. Two separate deletion alleles were made within exon 3 (*mvp*^*sq33*^; c.300_315del) and exon 7 (*mvp*^*sq43*^; c.758_762del) of the zebrafish *mvp* gene, leading to predicted frameshifts and truncated MVP protein products (p.L102Sfs13Ter and p.R253Hfs61Ter respectively). However, as with mouse *Mvp* mutants^23^, zebrafish *mvp* mutants had no observable phenotype whilst qPCR indicated variable transcript loss (Fig. 2a). In the absence of phenotype or antibody, we used SWATH to assess loss of zebrafish MVP protein. As expected, mutant 30hpf embryos from both *mvp* alleles showed substantially lower levels of MVP peptides across the entire protein compared to wild type embryos (Fig. 2b). MVP is the main constituent protein of Vault ribonucleoproteins, to which a diverse range of functions have been ascribed from antimicrobial to cell signalling^24^. Analysis of proteome changes in the mutants failed to identify coherent alterations in any particular signalling pathway or cell function (Supplementary Fig. S2), pointing to a potential role for MVP in homeostasis. Interestingly, the most highly under-expressed proteins in the *mvp*^*sq33/sq33*^ mutant (aside from MVP itself) was Histone2b (X1WDH8; Fig. 2C), a component of the nucleosome and not known to have any interaction with MVP. Upon further investigation, we determined that this mutant harboured a missense single-nucleotide polymorphism (SNP) in *h2b* (rs501382785; c.232 T > C), substituting the cysteine at position 78 to arginine (p.Cys78Arg) in the peptide IAGEASCLAHY. Subsequently, we used the Histone2b C78R-peptide to re-interrogate the wild type and *mvp*^*sq33/sq33*^ mutant SWATH analyses (Fig. 2d; Supplementary Fig. S3). Consequently, the C78R-peptide was detected in wild type and *mvp*^*sq33/sq33*^ mutant samples, indicating similar expression levels in both samples. This demonstrates that SNP representation in populations can be detected by SWATH, and that SWATH datasets can be interrogated retrospectively to test new or modified biological hypotheses, without any need for further MS data acquisition.

**Figure 3:**
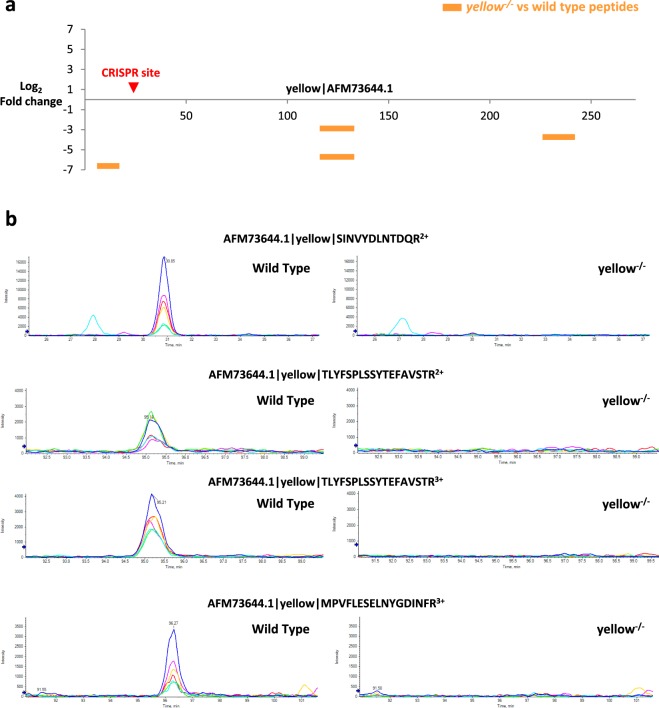
SWATH can validate protein silencing in non-model species without need for spectral library. (**a**) Predicted termination codon introduced by CRISPR-induced frameshift indicated by red arrow. SWATH quantification showed lower levels of yellow peptides (orange horizontal bars) in yellow^*−/−*^ samples as compared to wild type, indicating absence of the yellow protein in the mutant samples. (**b**) Representative extracted ion chromatograms (XICs) of the four quantified yellow precursors from wild type and yellow^*−/−*^ samples.

Typical SWATH workflows perform peptide identification by matching MSMS spectra against peptide fragment information (ion masses with their relative ion intensities and retention time) recorded in the reference spectral library. However, generating a reference spectral library may be challenging in some instances, especially in non-model species where a reference protein database or sequenced genomes are largely predicted. To overcome this, we explored the possibility of directly interrogating the SWATH data in a peptide-centric and library-independent manner using predicted fragment ion masses alone. Although the identification confidence may be slightly affected, this approach can nonetheless be very useful to verify gene editing in non-model organisms. To illustrate this, we analysed a Squinting Bush Brown butterfly (*Bicyclus anynana*) model with CRISPR-knockout of *yellow*, a gene in the melanin synthesis pathway that when mutated prevents the synthesis of dopa-melanin and alters the colour of the butterfly wings from brown to yellow^25^. Although the phenotype is visible, it is still important to link the absence of the Yellow protein to the phenotype. However, in addition to the lack of a protein reference database, only partial knowledge of the *B. anynana* Yellow protein sequence was available in the sequenced genome^26^. Nonetheless, we performed an *in silico* digestion of the partial yellow protein sequence and queried the resulting peptides against the SWATH data. Of the 19 theoretical tryptic peptides, nine were disregarded as they were unlikely to be detected in the MS detection mass range (Supplementary Fig. S4). Among the remaining, three peptides were matched in the SWATH data: one semi-tryptic (SINVYDLNTDQR) upstream of the CRISPR site, and the other two (TLYFSPLSSYTEFAVSTR and MPVFLESELNYGDINFR) downstream (Fig. 3a). In particular, peptide TLYFSPLSSYTEFAVSTR was identified at multiple charge states and eluting at the same retention time, further increasing the confidence of the peptide match. When comparing the extracted ion chromatograms of the four quantified precursors from wild type and *yellow*-knockout mutants, the absence of Yellow protein after CRISPR-induced knockout (Fig. 3b; Supplementary Figs S5, 6) was clearly observed as substantially lower peptide levels. From the above, we show that given some knowledge of the target protein sequence, SWATH can be used to confirm protein silencing in a library-independent manner.

In conclusion, we demonstrated the distinctive advantages of employing SWATH for confirming gene silencing at the protein level, which most importantly include the ability to obtain the proteome-level response in a single experiment. In all cases, we observed reduction of multiple peptides per targeted protein to non-detectable levels. As experiemnts were performed in a back-to-back manner, the confident detection of the peptides in the controls and not in the edited samples suggest that this is unlikely to be due to experimental noise. This approach provides evidence supporting and elaborating DNA genotyping of induced genetic lesions. Moreover, SWATH can be applied across different genome editing techniques and diverse organisms. In light of these, we propose that SWATH is an effective and direct approach for validation of genome-editing, and especially useful as an antibody-independent alternative for non-model organisms. Moreover, provided that we can detect the modified peptide/s, we expect that SWATH can be easily extended to detect other varieties of genome-editing (insertions, amino acid substitutions, fusions).

## Methods

### Mouse (*Mus musculus*) primary mammary gland epithelial cells

Murine primary mammary gland epithelial cells previously established from ANXA1^*+/−*^ and ANXA1^*−/−*^ mice^8,9^ were cultured in Dulbecco’s minimal essential medium (Thermo Fisher Scientific) supplemented with 10% FBS (Thermo Fisher Scientific) and 1% penicillin-streptomycin (Invitrogen), in a humidified incubator (37 °C, 5% CO_2_). Cells were harvested using trypsin and rinsed in PBS thrice before lysis in 50 mM triethylammonium bicarbonate, 8 M urea, 1% sodium deoxycholate (pH 8) at room temperature for 20 min. The resulting cell lysates were centrifuged at 21 100 × *g* for 20 min at 12 °C to remove cell debris. Protein quantification was performed using the Pierce 660 nm Protein Assay (Thermo Fisher Scientific), according to the manufacturer’s instructions.

### Zebrafish (*Danio rerio*) husbandry and generation of mutants

Zebrafish were housed at the Institute of Molecular and Cell Biology (IMCB) Zebrafish facility and were bred using standard crossing of the AB wild-type strain or nuclease generated mutants. Experimental methods and procedures were performed according to the National Advisory Committee for Laboratory Animal Research (NACLAR) Guidelines and approved by the Institutional Animal Care and Use Committee (IACUC) of the Agency for Science Technology and Research (A*STAR) Biological Resource Centre (Protocol #140924). CompoZr® Custom Zinc Finger Nucleases (ZFN) were purchased from Sigma-Aldrich to target the zebrafish *st14a* gene, which encodes the epithelial associated serine protease Matriptase1, with the left and right DNA binding Zinc finger domains fused to endonuclease portions of hetero-dimeric Fok1 Nuclease. The target site in exon 6 was 5′CAGTTCCAGCAGCACACGaagcaGCAGTGGATCAGGCTGTG3′, with the left and right Zinc Finger binding sites in uppercase, flanking the cut site in lowercase. Embryos derived from AB wild type in-crosses were injected with 1.7 nl of the supplied cocktail of both RNAs diluted to 50 ng/µl each RNA with Phenol Red tracer, using a Harvard Apparatus PLI-100 Pico-Injector. A selection of larvae was sequenced to check mutagenesis efficiency, and the remaining were raised. Offspring of injected individuals were sequenced for identifying founders. An allele which introduced a 5 bp insertion, *st14a*^*sq10*^, was used in this work.

CRISPR guide sequences targeting exons 3 and 7 of the zebrafish *mvp* gene (encoding for Major Vault Protein – the main component of Vault ribonucleoproteins) were identified using the ZiFiT online tool. Guide sequences for exon 3 and 7 were 5′GGAACGGGTCCTGGGCCAGG3′ and 5′GGTGGTCGGGATCGGCGCAC3′ respectively. Guide sites were cloned downstream of the T7 RNA priming site and upstream of the tracrRNA sequence located on the pDR274 plasmid^27^ by PCR of *BsaI* digested plasmid template, using primers modified with the guide sequences, and iProof™ (Bio-Rad). RNA was synthesised from *DraI* linearised plasmids using MEGAshortscript™ T7 Transcription Kit (Ambion), and then purified by isopropanol precipitation. Guide RNA was co-injected with Cas9 RNA into wild-type embryos as above, and screened for mutagenesis of the target site by sequencing. Founders carrying deletions in exon3 and exon7 were identified (alleles *mvp*^*sq33*^ and *mvp*^*sq43*^ respectively) and were expanded for use in this work.

### Protein extraction of 30 hours post fertilisation (hpf) zebrafish embryos and adult fin tissues

Chorions of 30hpf embryos were removed by partial digestion using 1 mg/ml pronase (Sigma Aldrich) in E3 buffer (5 mM NaCl, 0.17 mM KCl, 0.33 mM CaCl and 0.33 mM MgSO_4_) with swirling for 15 min at room temperature. Subsequently, pronase was completely removed by five quick washes with E3 to prevent degradation of high molecular weight proteins. Next, dechorionated embryos were transferred to calcium-free Ringers deyolking buffer (116 mM NaCl, 2.9 mM KCl and 5 mM HEPES, pH 7.2). Yolk sacs were disrupted under mechanical stress by passing the embryos through glass pipettes with tips narrowed by flame polishing. The yolks were then dissolved in the buffer by agitating the embryos in a thermomixer (1100 rpm) for five min at room temperature. Deyolked embryos were pelleted by centrifugation at 300 × *g* for two min at 4 °C. Four additional washing steps as above were carried out to ensure complete removal of the yolk.

Three biological replicates of deyolked embryos each were obtained for the wild type and *mvp* mutants. Each biological replicate consisted of pooled deyolked embryos (n = 300) lysed with ice-cold lysis buffer (50 mM Tris-HCl, 100 mM NaCl, 2% sodium deoxycholate, supplemented with 1x cOmplete, EDTA-free protease inhibitor cocktail (Roche)). Samples were incubated on ice for five min, and sonicated on ice at mid setting (Microson XL-2020), for three times, 15 sec pulses with 30 sec rest in between. The resulting lysates were incubated on ice for 15 mins before addition of Benzonase (Sigma Aldrich) followed by further incubation for 15 mins at room temperature with mild agitation. Debris was pelleted by centrifugation at 21 100 × *g* for 1 hr at 4 °C to obtain the cell lysates. Protein quantification was performed using the Pierce 660 nm Protein Assay (Thermo Fisher Scientific), according to the manufacturer’s instructions.

Three biological replicates of adult (3 months) zebrafish tail-fin clips each were collected for wild type and *st14a*^*−/−*^ samples. Each biological replicate consisted of pooled fin clips (n = 15) were obtained under anaesthesia with tricaine methanesulfonate (Sigma Aldrich), and washed twice in ice-cold phosphate-buffered saline (PBS) before transferring into ice-cold lysis buffer. Sonication lysis was carried out as described in the previous paragraph.

### qPCR analysis

qPCR on the MVP samples were carried out on Applied Biosystems StepOnePlus™ System using PrecisionFAST qPCR mastermix (Primerdesign^TM^). Reaction set-up was carried out as described by manufacturer and standard qPCR cycling conditions were applied. CT values for MVP were normalised against β-actin and represented as ΔCT. ΔCT of treatments was compared against that of the control sample and fold change was calculated from the value of 2^(−ΔΔCT)^. Forward primer 5′ - GACGCAGGATGAGGAATTGT and reverse primer 5′ – ACGTTTGGGTTTGTCTCCAG were used.

### Squinting Bush Brown butterfly (*Bicyclus anynana*) wings

Three biological replicates of wild type and *yellow*^*−/−*^ *B. anynana* wings^25^ dissected from pupae were frozen in liquid nitrogen and homogenised in ice-cold lysis buffer with a motorised micro-pestle on ice. The homogenised samples were then subject to sonication lysis as described in the earlier section.

### SWATH sample preparation

Equal amounts of total protein from wild type and mutant samples were reduced with 5 mM dithiothreitol (Sigma-Aldrich) for 30 min and alkylated with 10 mM iodoacetamide (Sigma-Aldrich) for 30 min in the dark. The proteins were then digested using lysyl endopeptidase (Wako Chemicals GmbH) at an enzyme-to-protein ratio of 1:100 at 37 °C overnight. Subsequently, trypsin (Promega) was added at an enzyme-to-protein ratio of 1:50 and further incubated at 37 °C for 8 hr. The peptide digests were acidified to 0.5% final concentration of formic acid (FA) to precipitate the sodium deoxycholate, and then desalted using the Empore C18-SD Extraction Disk Cartridge (3 M). The desalted peptides were dried using a vacuum concentrator and finally reconstituted in 2% acetonitrile (ACN), 0.1% FA for MS analysis. In addition, synthetic peptides from the iRT kit (Biognosys AG) were spiked in at 10% final concentration for retention time alignment.

### Mass spectrometry data acquisition

For reference spectral library generation, two µg of peptides from pooled wild type samples were injected (three technical replicates) and subjected to online reversed-phase (RP) LC separation on the ekspert nanoLC-425 system (Eksigent). The RP solvent A was 2% ACN, 0.1% FA and solvent B was 95% ACN, 0.1% FA. The peptides were first trapped on a precolumn (350 µm x 0.5 mm), then separated using an analytical column (75 µm x 15 cm), which were both packed with ChromXP C18CL 3 µm 120 Å phase (Eksigent). Peptide elution was performed using a two-step linear gradient, comprising 10–18% solvent B over 55 min, followed by 18–30% solvent B over 60 min at a flow rate of 300 nL/min. Eluted peptides were directly injected into the TripleTOF 6600 system (SCIEX) for MS-analysis using the data-dependent acquisition (DDA) mode. Precursor ions were selected from 400–1600 m/z with 250 ms accumulation per time-of-flight spectrum. Maximum of 50 precursors per cycle were selected for MS/MS analysis from each MS spectrum. MS/MS analysis of each precursor was performed in high sensitivity mode at 50 ms accumulation time across a mass range of 100–1800 m/z with dynamic exclusion for 15 s and rolling collision energy.

For SWATH data acquisition, online RP analyses were performed as described above, with the exception that 1 µg of peptides were injected from each wild type and mutant sample (three biological replicates each). Eluted peptides from the LC were analysed on the TripleTOF 6600 system in the SWATH-MS mode. Precursor ion data was collected from 400–1600 m/z with 50 ms accumulation time per spectrum. Variable window widths were used, specifying for a maximum of 120 (100 for *B. anynana*) variable windows across a precursor mass range of 400–1200 m/z, with a 1 Da window overlap and minimum window width of 4 Da (Supplementary Information). Rolling collision energy was enabled for each window with 5 eV spread. Fragment ion spectra were accumulated in high sensitivity mode for 25 ms over 100–1800 m/z mass range, resulting in a total cycle time of 3.1 s.

### Reference spectral library generation

Each sample-specific reference spectral library was generated by a combined search of the three technical replicates using the ProteinPilot 5.0 software (SCIEX). The spectra were identified by searching against either (i) zebrafish (*D. rerio*) UniProt Reference Proteome (2018 April release, 44 132 entries) or (ii) mouse (*M. musculus*) SwissProt Reference Proteome (2018 October release, 16 997 entries), using the “thorough search” mode in the Paragon search engine (v5.0.0.0). The following parameters were specified: cysteine alkylation by iodoacetamide, common biological modifications and detected protein threshold at 0.05. FDR analysis was performed against decoy reversed protein sequences generated from the respective databases.

### SWATH data processing

SWATH raw files were analysed against the reference ion library using the OneOmics workflow hosted on SCIEX CloudOS platform. Peptide and fragment ion selection, peak area extraction and scoring were performed as previously described^10^ with some modifications. For every detected protein, a maximum of ten peptides with six transitions each were automatically selected for peak area extraction. The following parameters for peak area extraction were used: 75 ppm ion library tolerance, 5 min extracted ion chromatogram (XIC) extraction window, considering only peptides with at least 99% confidence and less than 1% FDR, and excluding shared peptides. Retention time calibration was automatically performed using the endogenous peptides. Briefly, the top 100 peptides based on precursor intensity and confidence were selected across the entire RT elution range and then scored. Peptides with scores above the median score were then used for RT calibration. Subsequently, data normalisation (most likely ratio normalisation, MLR), and determination of fold change values and confidences were performed as reported previously^10^.

Global proteome responses were visualised as volcano plots using the ggplot2 package in R. Biological process network functional enrichment analysis was performed using MetaCore (Clarivate Analytics). Zebrafish proteins were mapped to their corresponding human orthologues before MetaCore analysis.

*B. anynana* SWATH datasets were analysed separately using a library-independent approach in the SWATH Qual Browser (SCIEX research-grade application in PeakView). The partial sequence of the yellow protein (AFM73644.1) was subjected to *in silico* tryptic digestion and peptides which were unlikely to be detected within MS precursor mass range were removed. Remaining peptides were then analysed against all 100 SWATH windows across the entire chromatographic elution time range in search for co-eluting theoretical fragment ions, accounting for multiple precursor charge states. Finally, the resulting matches were manually curated for fragment ion co-elution and MSMS spectra match against the theoretical peptide sequences. Peptide quantification was performed by extracting the transition peak areas using MultiQuant 3.0 (SCIEX) with the following parameters: 1.0 point Gaussian smoothing, 30 sec RT half window, 3.0 point minimum peak width, 40% noise percentage, 0 min baseline sub-window and 2.0 points peak splitting. Extracted peak areas for six transitions were summed for each peptide.

## Supplementary information

Supplementary Information

## Data Availability

The mass spectrometry proteomics data have been deposited to the ProteomeXchange Consortium (http://proteomecentral.proteomexchange.org) via the PRIDE partner repository^28^ with the dataset identifier PXD013176.
