# Supplementary material for: Tracking genome-editing and associated molecular perturbations by SWATH mass spectrometry
Source: Sci Rep. 2019 Oct 23;9:15240. doi: 10.1038/s41598-019-51612-z (PMC6811567; doi:10.1038/s41598-019-51612-z)
Supplement: Supplementary file 1 — Supplementary Information [file 41598_2019_51612_MOESM1_ESM.pdf]

Tracking genome-editing and associated molecular perturbations by SWATH mass spectrometry

Qifeng Lin, Larry W.L. Low, Adam Lau, Esther W.L. Chua, Yuji Matsuoka, Yilong Lian, Antónia Monteiro, Stephen Tate, Jayantha Gunaratne and Tom J. Carney

Supplementary Information

Supplementary note

List of raw/data files used for generating figures (deposited in ProteomeXchange, PXD013176)

Figure 1a

OneOmics_PeptideFoldChangeData-ANXA1-KOvsHet.xlsx

Figure 1b

OneOmics_PeptideFoldChangeData_St14avsWT.xlsx

Figure 1c, d

OneOmics_ProteinFoldChangeData_St14avsWT.xlsx

Figure 2b

OneOmics_PeptideFoldChangeData_MVPsq33vsWT.xlsx
OneOmics_PeptideFoldChangeData_MVPsq43vsWT.xlsx

Figure 2c

OneOmics_ProteinFoldChangeData_MVPsq33vsWT.xlsx

Figure 2d

160401_IS448_SWATH_2ug_AB1.wiff
160401_IS448_SWATH_2ug_MVP1.wiff

Figure 2e

MultiQuant_PrecursorQuantification_yellow.xlsx

Figure 2f

170728_G694_SWATH_1ug_WTB.wiff
170728_G694_SWATH_1ug_WTD.wiff
170728_G694_SWATH_1ug_WT3.wiff
170728_G694_SWATH_1ug_YA.wiff
170728_G694_SWATH_1ug_Y2.wiff
170728_G694_SWATH_1ug_Y3.wiff

Figure S1b, c

OneOmics_ProteinFoldChangeData-ANXA1-KOvsHet.xlsx

Figure S2a, c

OneOmics_ProteinFoldChangeData_MVPsq43vsWT.xlsx

Figure S2b

OneOmics_ProteinFoldChangeData_MVPsq33vsWT.xlsx

Figure S3

160401_IS448_SWATH_2ug_AB1.wiff
160401_IS448_SWATH_2ug_AB2.wiff
160401_IS448_SWATH_2ug_AB3.wiff
160401_IS448_SWATH_2ug_MVP1.wiff
160401_IS448_SWATH_2ug_MVP2.wiff
160401_IS448_SWATH_2ug_MVP3.wiff

Figure S5, 6

170728_G694_SWATH_1ug_WTB.wiff
170728_G694_SWATH_1ug_WTD.wiff
170728_G694_SWATH_1ug_WT3.wiff
170728_G694_SWATH_1ug_YA.wiff
170728_G694_SWATH_1ug_Y2.wiff
170728_G694_SWATH_1ug_Y3.wiff

Supplementary Figure Legends

Supplementary Figure S1

(a) Western blot analysis of ANXA1 in three biological replicates of ANXA1$^{+/-}$ and ANXA1$^{-/-}$ samples. β-actin (ACTB) was used as a loading control. (b) Volcano plot visualisation of accompanying proteome changes in response to ANXA1 knockout, showing 168 over-expressed and 299 under-expressed proteins with cut-off criteria of fold change > 1.5 and fold change confidence > 0.7. (c) Functional analysis of top ten biological process networks significantly enriched in differentially expressed proteins.

Supplementary Figure S2

(a) Volcano plot visualisation of accompanying proteome changes in response to loss of MVP in the *mvp*$^{sq43/\,sq43}$ mutant, showing 65 over-expressed and 93 under-expressed proteins with cut-off criteria of fold change > 1.5 and fold change confidence > 0.7. Top ten biological process networks which are significantly enriched in differentially expressed proteins in (b) *mvp*$^{sq33/\,sq43}$ and (c) *mvp*$^{sq43/\,sq43}$ mutants compared to wild type.

Supplementary Figure S3

(a) XICs of canonical H2b peptide IAGEASCLAHY in three biological replicates of wild type and *mvp*$^{sq33/\,sq33}$ each. (b) XICs of SNP-variant C78R-H2b peptide IAGEASRLAHY in the same three biological replicates of wild type and *mvp*$^{sq33/\,sq33}$. (c) Summed SWATH spectrum for the most likely peak group for SNP-variant peptide IAGEASRLAHY, from experiment covering mass range 590.9 – 596.1 m/z and time 45.101 min.

Supplementary Figure S4

Partial sequence of *B. anynana* yellow protein, with premature termination codon introduced at indicated CRISPR site (red arrow). Peptides indicated in grey were disregarded due to incompatibility with MS detection range, while those indicated in green were detected and quantified in the SWATH data.

Supplementary Figure S5

(a) XICs and quantifications of yellow TLYFSPLSSYTEFAVSTR peptide in three biological replicates of wild type and yellow$^{-/-}$ each, in triple- and double-charged forms. Peptide quantification determined from summed peak areas of six transitions. (b) Summed SWATH spectrum for the most likely peak group for peptide TLYFSPLSSYTEFAVSTR, from experiment covering mass range 688.7 - 694.5 m/z and time 94.782 – 95.659 min.

Supplementary Figure S6

(a) XICs and quantifications of yellow SINVYDLNTDQR peptide in three biological replicates of wild type and yellow$^{-/-}$ each. Peptide quantification determined from summed peak areas of six transitions. (b) Summed SWATH spectrum for the most likely peak group for peptide SINVYDLNTDQR, from experiment covering mass range 715.1 – 720.3 m/z and time 30.086 – 31.676 min. (c) XICs and quantifications of yellow MPVFLESELNYGDINFR peptide in three biological replicates of wild type and yellow$^{-/-}$ each. Peptide quantification determined from summed peak areas of six transitions. (d) Summed SWATH spectrum for the most likely peak group for peptide MPVFLESELNYGDINFR, from experiment covering mass range 680.3 – 685.5 m/z and time 95.878 – 96.755 min.

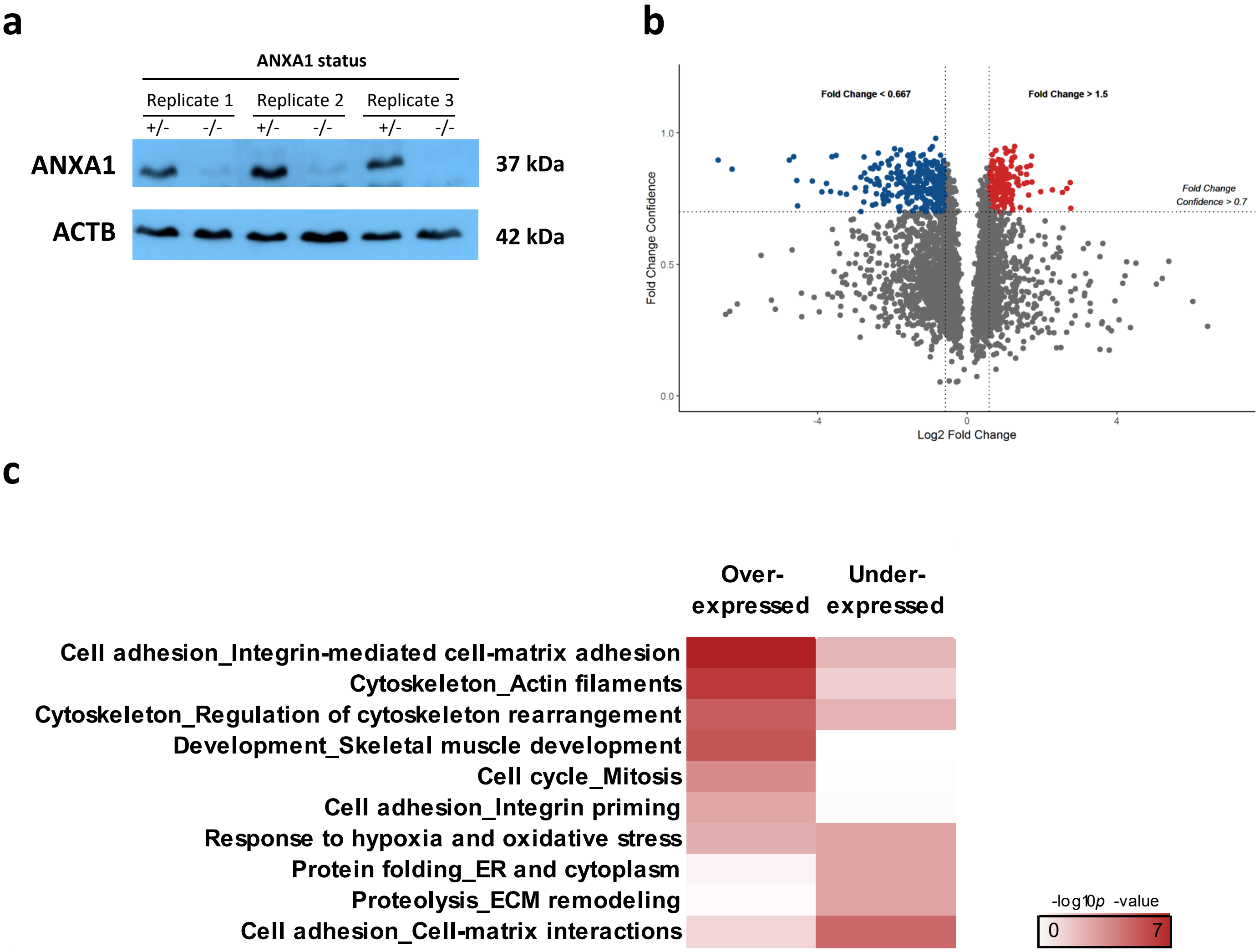

Supplementary Figure S1

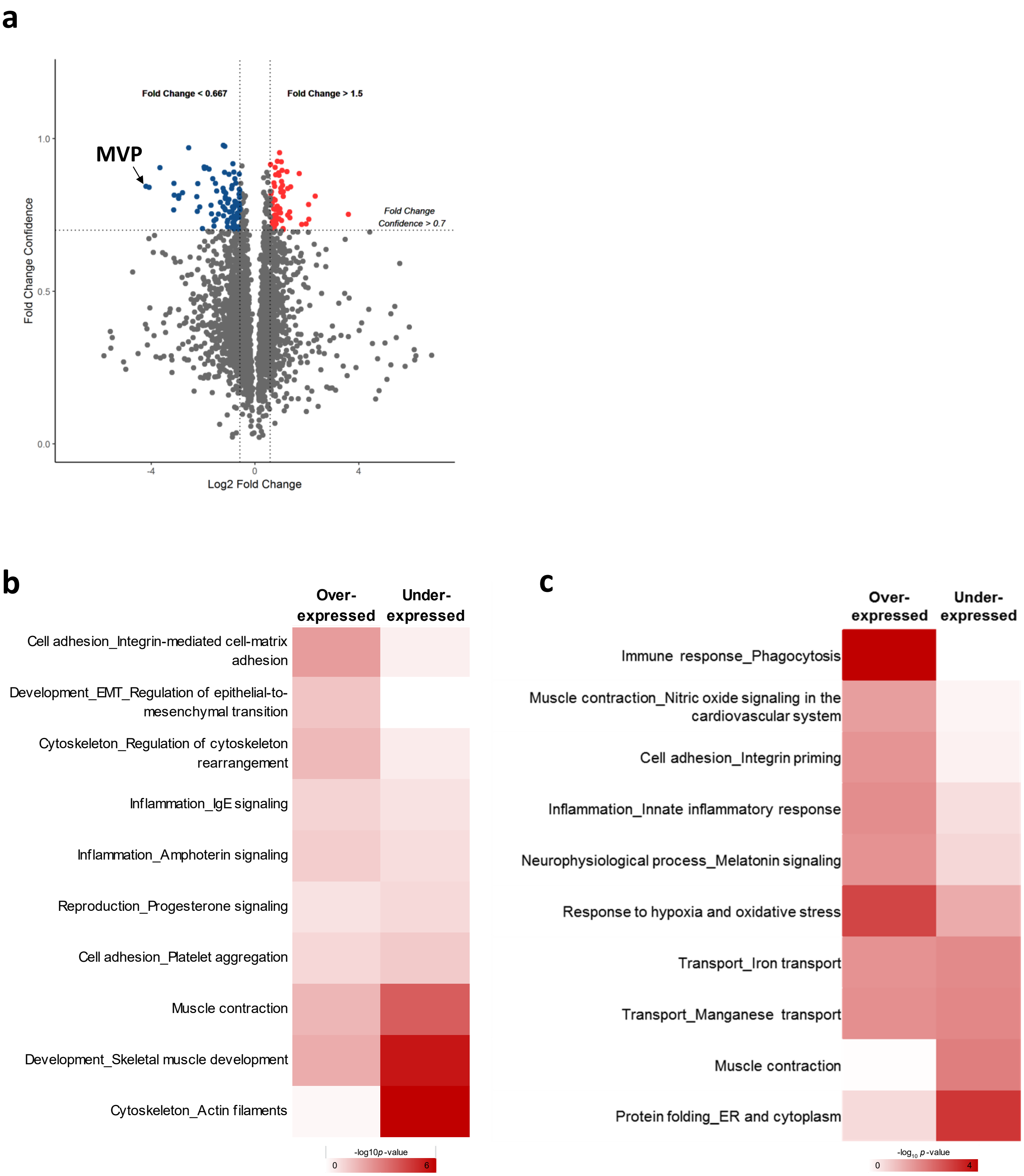

Supplementary Figure S2

a

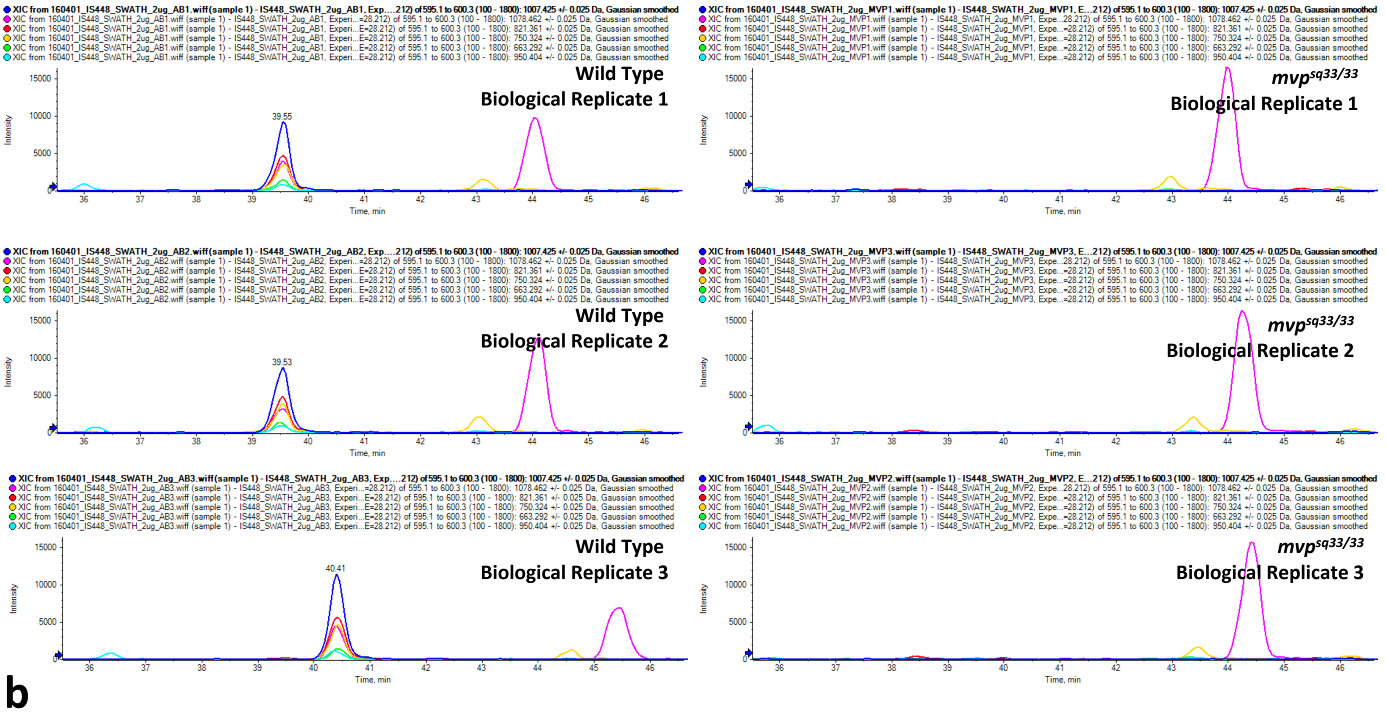

b

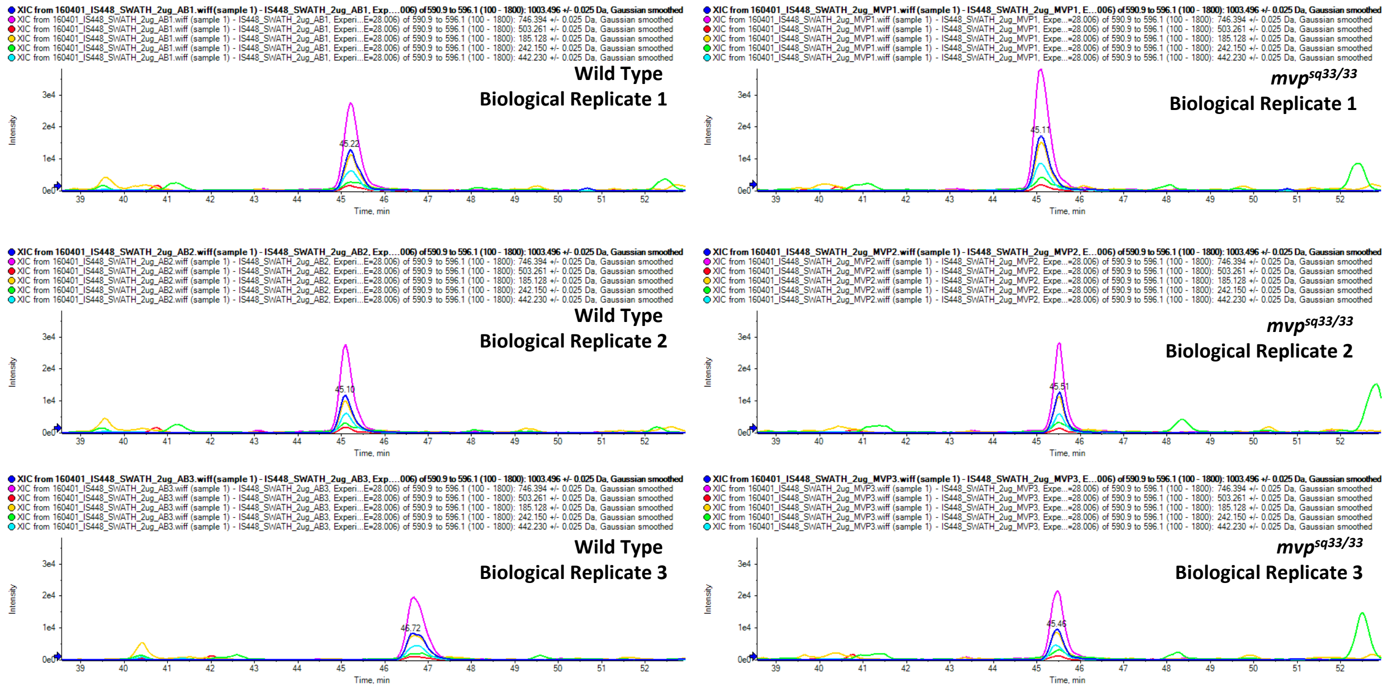

c

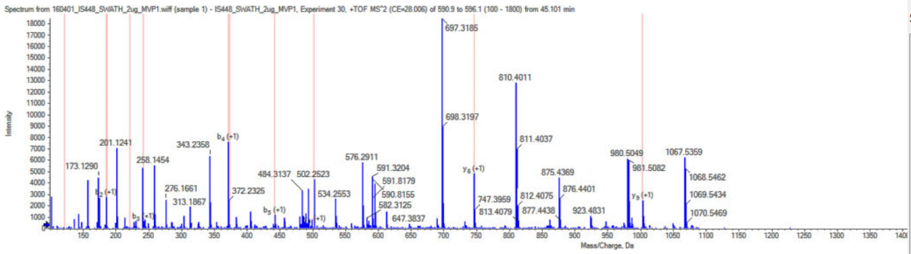

Precursor charge: 2 Target fragment charge: 1 Calculate

Sequence: IAGEASRLAHY

Table List Theoretical precursor m/z: 594.3120

Symbol	Res. Mass	# (N)	b	y	# (C)
I	113.08406	1	114.09134	*1187.61675*	11
A	71.03711	2	**185.12845**	1074.53268	10
G	57.02146	3	**242.14992**	***1003.49557***	9
E	129.04259	4	**371.19251**	946.47411	8
A	71.03711	5	**442.22963**	817.43151	7
S	87.03203	6	529.26165	**746.39440**	6
R	156.10111	7	*685.36276*	659.36237	5
L	113.08406	8	798.44683	**503.26126**	4
A	71.03711	9	869.48394	390.17720	3
H	137.05891	10	1006.54285	319.14008	2
Y	163.06333	11	1169.60618	182.08117	1

Supplementary Figure S3

>AFM73644.1 yellow, partial [Bicyclus anynana]

CRISPR site

NVCPYSINVYDLNTDQR IRR YVFRPEDIVSTTFIANIALDEGLTCDDTFAYFSDELGYGLIAYSWEQNK
SWR FSHSYFMPDPLVGDFNIAGLNFQWGAEGIFGISASPMGADGFR TLYFSPLSSYTEFAVSTR ILR
DETK VTGSYK DFK VVGTR GR DTHTTSK VMDVTGVQLFNLIDQNAIGCWSTNFALKPQNIAVVDK
DDVGLVFPCDIK IDEGR NVWVISDR MPVFLESELNYGDINFR IYVGSVDSLTQGTVCETSPPVTTPIIPTSP

Supplementary Figure S4

a

AFM73644.1|yellow|TLYFSPLSSYTEFAVSTR^{3+}

Wild Type Biological Replicate 1	Summed peak areas	*yellow*$^{-/-}$ Biological Replicate 1	Summed peak areas
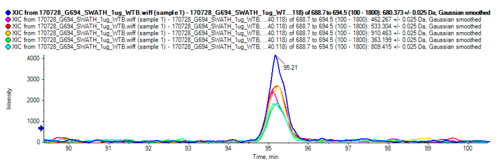	4.77E5	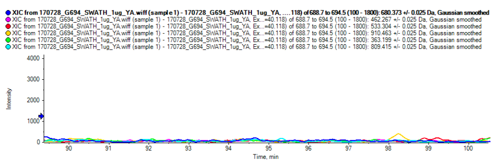	1.16E4
**Wild Type Biological Replicate 2**	**Summed peak areas**	***yellow*$^{-/-}$ Biological Replicate 2**	**Summed peak areas**
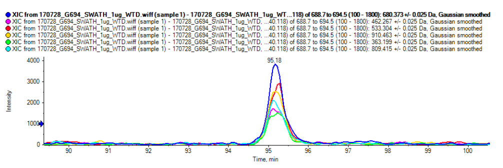	4.48E5	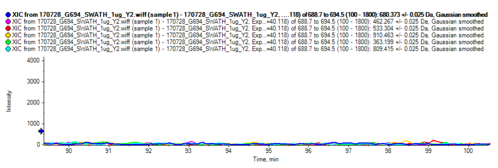	3.57E3
**Wild Type Biological Replicate 3**	**Summed peak areas**	***yellow*$^{-/-}$ Biological Replicate 3**	**Summed peak areas**
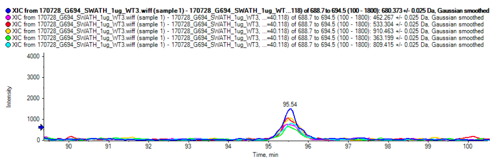	1.85E5	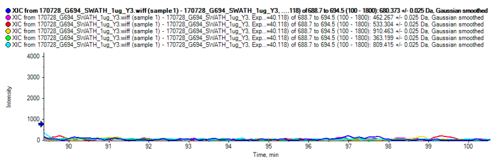	6.08E3

AFM73644.1|yellow|TLYFSPLSSYTEFAVSTR^{2+}

Wild Type Biological Replicate 1	Summed peak areas	*yellow*$^{-/-}$ Biological Replicate 1	Summed peak areas
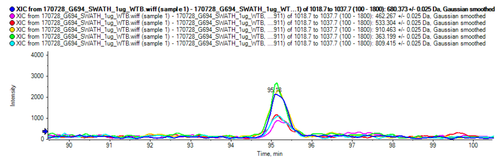	3.21E5	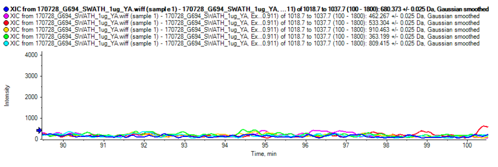	3.45E4
**Wild Type Biological Replicate 2**	**Summed peak areas**	***yellow*$^{-/-}$ Biological Replicate 2**	**Summed peak areas**
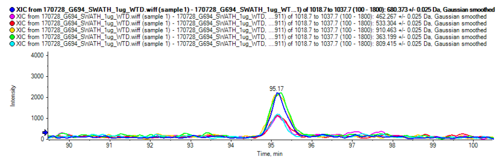	3.01E5	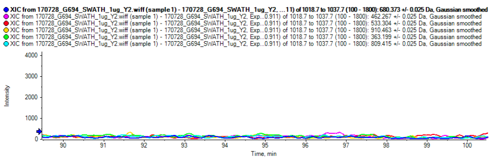	3.30E4
**Wild Type Biological Replicate 3**	**Summed peak areas**	***yellow*$^{-/-}$ Biological Replicate 3**	**Summed peak areas**
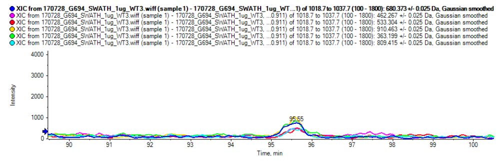	1.32E5	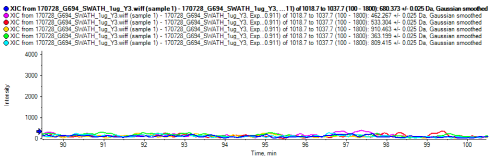	3.47E4

b

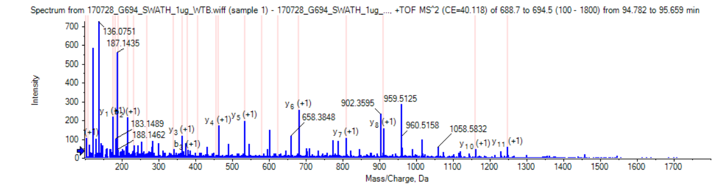

Symbol	Res. Mass	# (N)	b	y	# (C)
T	101.04768	1	102.05496	2069.02260	18
L	113.08406	2	215.13902	1967.97492	17
Y	163.06333	3	378.20235	1854.89086	16
F	147.06841	4	525.27076	1691.82753	15
S	87.03203	5	612.30279	1544.75912	14
P	97.05276	6	709.35555	1457.72709	13
L	113.08406	7	822.43962	1360.67432	12
S	87.03203	8	909.47165	1247.59026	11
S	87.03203	9	996.50368	1160.55823	10
Y	163.06333	10	1159.56700	1073.52620	9
T	101.04768	11	1260.61468	910.46287	8
E	129.04259	12	1389.65728	809.41519	7
F	147.06841	13	1536.72569	680.37260	6
A	71.03711	14	1607.76280	533.30419	5
V	99.06841	15	1706.83122	462.26707	4
S	87.03203	16	1793.86325	363.19866	3
T	101.04768	17	1894.91092	276.16663	2
R	156.10111	18	2051.01204	175.11895	1

Supplementary Figure S5

a

AFM73644.1|yellow|SINVYDLNTDQR^{2+}

Wild Type Biological Replicate 1	Summed peak areas	*yellow*$^{-/-}$ Biological Replicate 1	Summed peak areas
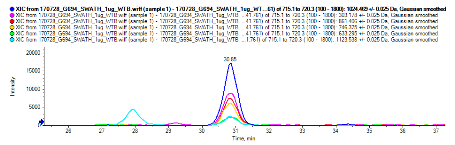	1.33E6	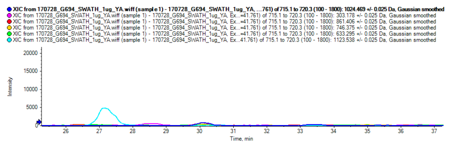	1.32E4
**Wild Type Biological Replicate 2**	**Summed peak areas**	***yellow*$^{-/-}$ Biological Replicate 2**	**Summed peak areas**
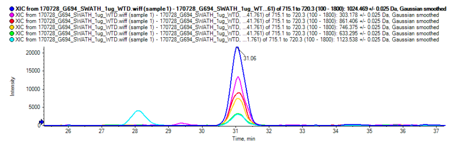	1.70E6	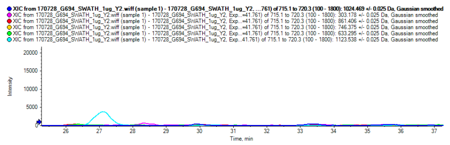	1.08E4
**Wild Type Biological Replicate 3**	**Summed peak areas**	***yellow*$^{-/-}$ Biological Replicate 3**	**Summed peak areas**
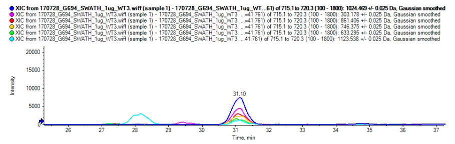	6.26E5	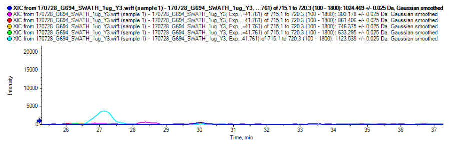	1.30E4

b

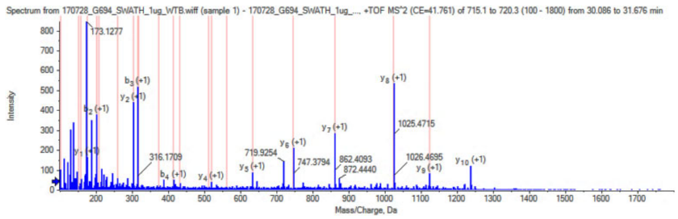

	Symbol	Res. Mass	# (N)	b	y	# (C)
▶	S	87.03203	1	88.03930	1437.69685	12
	I	113.08406	2	*201.12337*	1350.66482	11
	N	114.04293	3	**315.16630**	**1237.58076**	10
	V	99.06841	4	**414.23471**	**1123.53783**	9
	Y	163.06333	5	577.29804	**1024.46942**	8
	D	115.02694	6	692.32498	**861.40609**	7
	L	113.08406	7	805.40905	**746.37914**	6
	N	114.04293	8	919.45197	**633.29508**	5
	T	101.04768	9	1020.49965	**519.25215**	4
	D	115.02694	10	1135.52660	418.20447	3
	Q	128.05858	11	1263.58517	**303.17753**	2
	R	156.10111	12	1419.68628	**175.11895**	1

c

AFM73644.1|yellow|MPVFLESELNYGDINFR^{3+}

Wild Type Biological Replicate 1	Summed peak areas	*yellow*$^{-/-}$ Biological Replicate 1	Summed peak areas
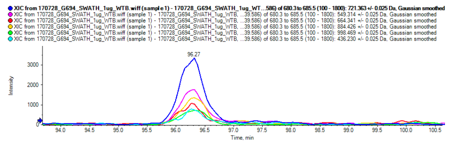	2.78E5	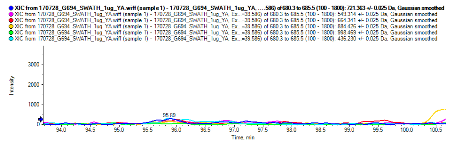	3.80E4
**Wild Type Biological Replicate 2**	**Summed peak areas**	***yellow*$^{-/-}$ Biological Replicate 2**	**Summed peak areas**
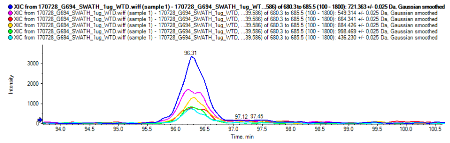	2.67E5	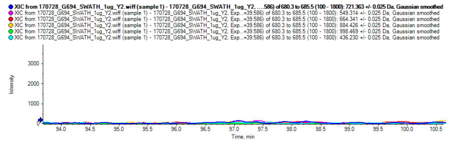	3.54E3
**Wild Type Biological Replicate 3**	**Summed peak areas**	***yellow*$^{-/-}$ Biological Replicate 3**	**Summed peak areas**
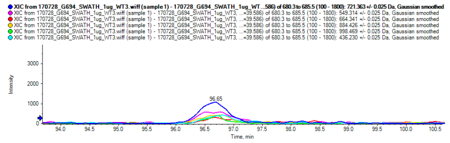	1.19E5	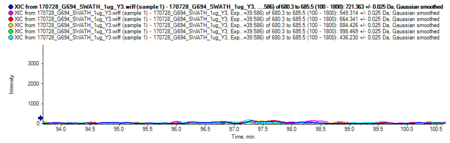	8.47E3

d

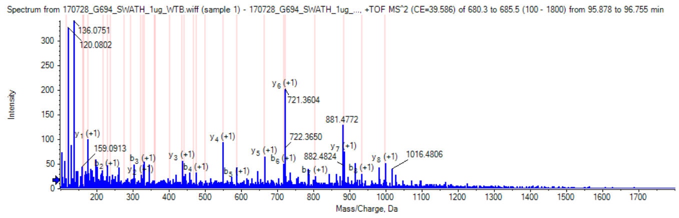

	Symbol	Res. Mass	# (N)	b	y	# (C)
▶	M	131.04049	1	132.04776	2043.98444	17
	P	97.05276	2	*229.10053*	1912.94396	16
	V	99.06841	3	**328.16894**	1815.89119	15
	F	147.06841	4	**475.23735**	1716.82278	14
	L	113.08406	5	**588.32142**	1569.75436	13
	E	129.04259	6	**717.36401**	1456.67030	12
	S	87.03203	7	**804.39604**	*1327.62771*	11
	E	129.04259	8	**933.43863**	1240.59568	10
	L	113.08406	9	1046.52270	1111.55309	9
	N	114.04293	10	1160.56562	**998.46902**	8
	Y	163.06333	11	1323.62895	**884.42609**	7
	G	57.02146	12	1380.65042	**721.36276**	6
	D	115.02694	13	1495.67736	**664.34130**	5
	I	113.08406	14	1608.76142	**549.31436**	4
	N	114.04293	15	1722.80435	**436.23029**	3
	F	147.06841	16	1869.87277	**322.18737**	2
	R	156.10111	17	2025.97388	**175.11895**	1

Supplementary Figure S6

SWATH variable window setup for mouse ANXA1 study

399.5	405.9	5
404.9	411.3	5
410.3	416.7	5
415.7	421.5	5
420.5	426.3	5
425.3	431.1	5
430.1	435.9	5
434.9	440.7	5
439.7	444.9	5
443.9	449.1	5
448.1	453.9	5
452.9	458.1	5
457.1	461.7	5
460.7	465.9	5
464.9	470.1	5
469.1	474.3	5
473.3	477.9	5
476.9	482.1	5
481.1	486.3	5
485.3	489.9	5
488.9	494.1	5
493.1	497.7	5
496.7	501.9	5
500.9	505.5	5
504.5	509.7	5
508.7	513.3	5
512.3	517.5	5
516.5	521.1	5
520.1	524.7	5
523.7	528.9	5
527.9	532.5	5
531.5	536.1	5
535.1	539.7	5
538.7	543.9	5
542.9	547.5	5
546.5	551.1	5
550.1	554.7	5
553.7	558.9	5
557.9	562.5	5
561.5	566.1	5
565.1	569.7	5
568.7	573.3	5
572.3	577.5	5
576.5	581.1	5
580.1	584.7	5
583.7	588.3	5
587.3	591.9	5
590.9	595.5	5
594.5	599.7	5
598.7	603.3	5
602.3	606.9	5
605.9	610.5	5
609.5	614.7	5
613.7	618.3	5
617.3	621.9	5
620.9	625.5	5
624.5	629.1	5
628.1	633.3	5
632.3	636.9	5
635.9	640.5	5
639.5	644.7	5
643.7	648.3	5
647.3	652.5	5
651.5	656.7	5
655.7	660.9	5
659.9	665.1	5
664.1	669.3	5
668.3	673.5	5
672.5	677.7	5
676.7	681.9	5
680.9	686.1	5
685.1	690.9	5
689.9	695.1	5
694.1	699.9	5
698.9	704.7	5
703.7	710.1	5
709.1	714.9	5
713.9	720.3	5
719.3	725.7	5
724.7	731.1	5
730.1	736.5	5
735.5	741.9	5
740.9	747.3	5
746.3	753.3	5
752.3	758.7	5
757.7	764.7	5
763.7	770.7	5
769.7	776.7	5
775.7	783.3	5
782.3	789.3	5
788.3	795.9	5
794.9	802.5	5
801.5	809.7	5
808.7	816.3	5
815.3	823.5	5
822.5	830.7	5
829.7	838.5	5
837.5	846.3	5
845.3	854.1	5
853.1	861.9	5
860.9	869.7	5
868.7	878.1	5
877.1	886.5	5
885.5	895.5	5
894.5	904.5	5
903.5	914.7	5
913.7	924.9	5
923.9	935.7	5
934.7	947.7	5
946.7	960.9	5
959.9	975.9	5
974.9	991.5	5
990.5	1008.9	5
1007.9	1028.1	5
1027.1	1049.7	5
1048.7	1073.1	5
1072.1	1097.7	5
1096.7	1125.3	5
1124.3	1158.3	5
1157.3	1199.7	5

SWATH variable window setup for zebrafish
St14a study

399.5	405.9	5
404.9	410.7	5
409.7	416.1	5
415.1	422.1	5
421.1	427.5	5
426.5	432.9	5
431.9	438.3	5
437.3	443.7	5
442.7	449.1	5
448.1	453.9	5
452.9	458.7	5
457.7	464.1	5
463.1	468.9	5
467.9	473.1	5
472.1	477.9	5
476.9	482.7	5
481.7	486.9	5
485.9	491.7	5
490.7	495.9	5
494.9	500.7	5
499.7	504.9	5
503.9	509.7	5
508.7	513.9	5
512.9	518.7	5
517.7	522.9	5
521.9	527.1	5
526.1	531.9	5
530.9	536.1	5
535.1	540.3	5
539.3	544.5	5
543.5	548.7	5
547.7	552.9	5
551.9	557.1	5
556.1	561.3	5
560.3	565.5	5
564.5	569.7	5
568.7	573.9	5
572.9	578.1	5
577.1	582.3	5
581.3	586.5	5
585.5	590.1	5
589.1	594.3	5
593.3	598.5	5
597.5	602.7	5
601.7	606.9	5
605.9	611.1	5
610.1	615.3	5
614.3	619.5	5
618.5	623.7	5
622.7	627.3	5
626.3	631.5	5
630.5	635.7	5
634.7	639.9	5
638.9	643.5	5
642.5	647.7	5
646.7	651.9	5
650.9	656.1	5
655.1	660.3	5
659.3	664.5	5
663.5	668.7	5
667.7	672.9	5
671.9	677.1	5
676.1	681.3	5
680.3	685.5	5
684.5	689.7	5
688.7	693.9	5
692.9	698.1	5
697.1	702.3	5
701.3	706.5	5
705.5	711.3	5
710.3	715.5	5
714.5	720.3	5
719.3	725.1	5
724.1	729.9	5
728.9	734.7	5
733.7	740.1	5
739.1	744.9	5
743.9	750.3	5
749.3	755.7	5
754.7	761.1	5
760.1	766.5	5
765.5	771.9	5
770.9	777.9	5
776.9	783.9	5
782.9	789.9	5
788.9	795.9	5
794.9	801.9	5
800.9	807.9	5
806.9	814.5	5
813.5	820.5	5
819.5	827.1	5
826.1	834.3	5
833.3	840.9	5
839.9	848.1	5
847.1	855.3	5
854.3	862.5	5
861.5	869.7	5
868.7	877.5	5
876.5	885.3	5
884.3	893.1	5
892.1	902.1	5
901.1	910.5	5
909.5	920.1	5
919.1	929.7	5
928.7	939.9	5
938.9	950.7	5
949.7	962.1	5
961.1	974.7	5
973.7	987.3	5
986.3	1001.1	5
1000.1	1014.9	5
1013.9	1029.3	5
1028.3	1044.9	5
1043.9	1061.1	5
1060.1	1078.5	5
1077.5	1097.1	5
1096.1	1117.5	5
1116.5	1141.5	5
1140.5	1168.5	5
1167.5	1199.7	5

SWATH variable window setup for zebrafish MVP study

399.5	423.3	5
422.3	441.3	5
440.3	452.1	5
451.1	461.1	5
460.1	468.9	5
467.9	476.7	5
475.7	483.9	5
482.9	490.5	5
489.5	497.1	5
496.1	503.1	5
502.1	509.1	5
508.1	514.5	5
513.5	520.5	5
519.5	525.9	5
524.9	531.3	5
530.3	536.7	5
535.7	542.1	5
541.1	547.5	5
546.5	552.3	5
551.3	557.1	5
556.1	561.3	5
560.3	566.1	5
565.1	570.9	5
569.9	575.1	5
574.1	579.3	5
578.3	583.5	5
582.5	587.7	5
586.7	591.9	5
590.9	596.1	5
595.1	600.3	5
599.3	604.5	5
603.5	608.1	5
607.1	612.3	5
611.3	616.5	5
615.5	620.1	5
619.1	624.3	5
623.3	627.9	5
626.9	632.1	5
631.1	635.7	5
634.7	639.9	5
638.9	643.5	5
642.5	647.7	5
646.7	651.3	5
650.3	655.5	5
654.5	659.1	5
658.1	663.3	5
662.3	667.5	5
666.5	671.1	5
670.1	675.3	5
674.3	678.9	5
677.9	683.1	5
682.1	687.3	5
686.3	690.9	5
689.9	695.1	5
694.1	698.7	5
697.7	702.9	5
701.9	706.5	5
705.5	710.7	5
709.7	714.9	5
713.9	719.1	5
718.1	723.3	5
722.3	726.9	5
725.9	731.1	5
730.1	735.3	5
734.3	739.5	5
738.5	743.7	5
742.7	747.9	5
746.9	752.1	5
751.1	756.3	5
755.3	760.5	5
759.5	765.3	5
764.3	769.5	5
768.5	774.3	5
773.3	778.5	5
777.5	783.3	5
782.3	788.1	5
787.1	792.9	5
791.9	797.7	5
796.7	802.5	5
801.5	807.3	5
806.3	812.7	5
811.7	817.5	5
816.5	822.9	5
821.9	828.3	5
827.3	833.1	5
832.1	838.5	5
837.5	843.9	5
842.9	849.3	5
848.3	855.3	5
854.3	860.7	5
859.7	866.1	5
865.1	872.1	5
871.1	878.1	5
877.1	884.1	5
883.1	890.1	5
889.1	896.7	5
895.7	903.3	5
902.3	910.5	5
909.5	917.7	5
916.7	925.5	5
924.5	933.9	5
932.9	942.3	5
941.3	951.3	5
950.3	960.3	5
959.3	970.5	5
969.5	980.7	5
979.7	992.1	5
991.1	1003.5	5
1002.5	1015.5	5
1014.5	1028.1	5
1027.1	1041.9	5
1040.9	1056.3	5
1055.3	1071.3	5
1070.3	1086.9	5
1085.9	1102.5	5
1101.5	1119.3	5
1118.3	1136.7	5
1135.7	1155.3	5
1154.3	1176.3	5
1175.3	1199.7	5

SWATH variable window setup for butterfly
Yellow study

399.5	437.7	5
436.7	456.9	5
455.9	471.3	5
470.3	482.7	5
481.7	492.9	5
491.9	501.9	5
500.9	510.3	5
509.3	518.1	5
517.1	524.7	5
523.7	531.9	5
530.9	537.9	5
536.9	543.9	5
542.9	549.9	5
548.9	555.3	5
554.3	560.7	5
559.7	566.1	5
565.1	570.9	5
569.9	575.7	5
574.7	580.5	5
579.5	585.3	5
584.3	590.1	5
589.1	594.9	5
593.9	599.1	5
598.1	603.9	5
602.9	608.1	5
607.1	612.3	5
611.3	617.1	5
616.1	621.3	5
620.3	625.5	5
624.5	629.7	5
628.7	634.5	5
633.5	638.7	5
637.7	642.9	5
641.9	647.1	5
646.1	651.3	5
650.3	655.5	5
654.5	660.3	5
659.3	664.5	5
663.5	668.7	5
667.7	672.9	5
671.9	677.1	5
676.1	681.3	5
680.3	685.5	5
684.5	689.7	5
688.7	694.5	5
693.5	698.7	5
697.7	702.9	5
701.9	707.1	5
706.1	711.9	5
710.9	716.1	5
715.1	720.3	5
719.3	725.1	5
724.1	729.9	5
728.9	734.1	5
733.1	738.9	5
737.9	743.7	5
742.7	748.5	5
747.5	753.3	5
752.3	758.1	5
757.1	763.5	5
762.5	768.3	5
767.3	773.7	5
772.7	779.1	5
778.1	784.5	5
783.5	789.9	5
788.9	795.3	5
794.3	801.3	5
800.3	806.7	5
805.7	812.7	5
811.7	818.1	5
817.1	824.1	5
823.1	830.7	5
829.7	836.7	5
835.7	842.7	5
841.7	849.3	5
848.3	855.9	5
854.9	863.1	5
862.1	869.7	5
868.7	876.9	5
875.9	884.7	5
883.7	892.5	5
891.5	900.3	5
899.3	908.7	5
907.7	916.5	5
915.5	925.5	5
924.5	934.5	5
933.5	944.1	5
943.1	953.7	5
952.7	964.5	5
963.5	975.9	5
974.9	989.1	5
988.1	1003.5	5
1002.5	1019.7	5
1018.7	1037.7	5
1036.7	1056.9	5
1055.9	1078.5	5
1077.5	1101.9	5
1100.9	1128.9	5
1127.9	1160.1	5
1159.1	1199.7	5
